# Neglected Tropical Diseases and Female Infertility: Possible Pathophysiological Mechanisms

**DOI:** 10.1155/jotm/2126664

**Published:** 2025-04-30

**Authors:** Moses Agbomhere Hamed, Olabanji Ahmed Surakat, Victor Olukayode Ekundina, Kabirat Bolajoko Jimoh, Adetomiwa Ezekiel Adeogun, Nafisat Omolola Akanji, Oluwafemi Joshua Babalola, Patrick Chukwunonso Eya

**Affiliations:** ^1^Department of Neuroendocrinology, The Brainwill Laboratory, Osogbo, Osun State, Nigeria; ^2^Department of Medical Laboratory Science, Afe Babalola University, Ado-Ekiti, Ekiti State, Nigeria; ^3^Department of Zoology, Faculty of Basic and Applied Sciences, Osun State University, Osogbo, Osun State, Nigeria; ^4^Department of Physiology, Faculty of Basic Medical Sciences, Osun State University, Osogbo, Osun State, Nigeria; ^5^Department of Physiology, Ladoke Akintola University of Technology, Ogbomoso, Oyo State, Nigeria; ^6^Department of Physiology, University of Ibadan, Ibadan, Oyo State, Nigeria; ^7^Department of Environmental Health Science, National Open University of Nigeria, Jabi, Abuja, Nigeria

**Keywords:** bacteria, female infertility, fungi, neglected tropical diseases, parasite, viruses

## Abstract

Battling female infertility has posed a global challenge, where neglected tropical diseases (NTDs) are nonetheless a notable contributing factor. NTDs affect a variety of diseases, often of a chronic nature, which are often cited as some of the most lethal diseases operating against the most economically disadvantaged populations across the globe. The various causative agents for NTDs have been documented and could originate from a myriad of sources—from bacteria, fungi and viruses to ecto- and endoparasitic species—including but not limited to helminths and protozoa. This paper will seek to describe how NTDs influence female reproductive health, together with likely mediators. While these diseases have curable forms, their effects have gone well beyond female infertility, to major pain, disability and even mortality, particularly in poorer countries, thus causing economic hardship, reduced productivity and a pool of social stigma. NTDs adversely affect female reproductive functions through multiple mechanisms, including ROS-sensitive signalling, depression of steroidogenic markers and promotion of apoptosis. The effects also may reflect their influence on ovarian histomorphology, consequently resulting in female infertility. Current-directed studies, however, suggest a potential benefit in combining drugs for the most common NTDs as a deterrent to possible female infertility endowed by NTD infection. Nonetheless, further clinical investigations will be instrumental in elucidating the probable preventive value of combination drugs as adjuvant therapy to NTDs infections. This will provide comprehensive insight into the pathophysiological and molecular basis for the impairment of female fertility brought about by NTDs, leading to the development of preventive models to curb the adverse effects of NTDs on female reproductive health. Therefore, attention should be given to providing the right, timely and effective mode of treatment for NTDs-related female infertility.

## 1. Introduction

Infertility refers to a condition characterised by an inability to conceive and successfully carry a pregnancy to term. In contradistinction to sterility, infertility is not an inherently irreversible condition [1]. The prevalence of infertility in women is estimated to be about 13%, whereas in males, it is estimated to be roughly 10%. The primary factors contributing to female infertility are anovulation, fallopian tube pathology, pelvic adhesions, endometriosis and unexplained infertility [2]. The implications of infertility include social ramifications, individual distress, psychiatric problems [3, 4], and sexual dysfunctions [5].

**Figure 1:**
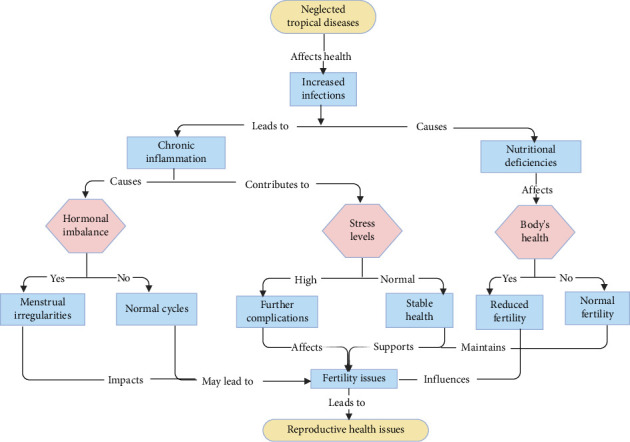
Flowchart on the impacts of neglected tropical diseases on female fertility.

Infertility issues are associated with several adverse consequences [6–8]. One notable consequence of infertility is the emergence of neglected tropical diseases (NTDs). The occurrence of congenital infections with certain pathogens associated with neural tube defects has been shown to be frequent (Figure 1). Congenital toxoplasmosis and malaria are widely recognised as prominent instances [9, 10]. However, recent evidence suggests that congenital Chagas disease is also prevalent among pregnant mothers who test positive for the infection, especially those exhibiting parasitaemia [11, 12]. The occurrence of congenital leishmaniasis has been shown in previous studies [13, 14], as has the transmission of hookworm and strongyloidiasis via breastfeeding [15, 16].

The NTDs include a collection of mostly chronic ailments that have been recognised as significant contributors to morbidity and death (Table 1), particularly among the most economically disadvantaged groups worldwide. Additionally, research has shown a correlation between these diseases and the perpetuation of poverty [36, 37]. The collective impact of NTDs has led to their classification as one of four major disease groups, alongside human immunodeficiency virus/acquired immunodeficiency virus (HIV/AIDS), tuberculosis (TB) and malaria [37]. NTDs are responsible for more than 500,000 fatalities each year (Table 1) and have been shown to cause a higher burden of disability-adjusted life years lost, compared to malaria and TB, as assessed in 2007 [6]. In this context, a wide range of infections might fall under consideration.

**Figure 2:**
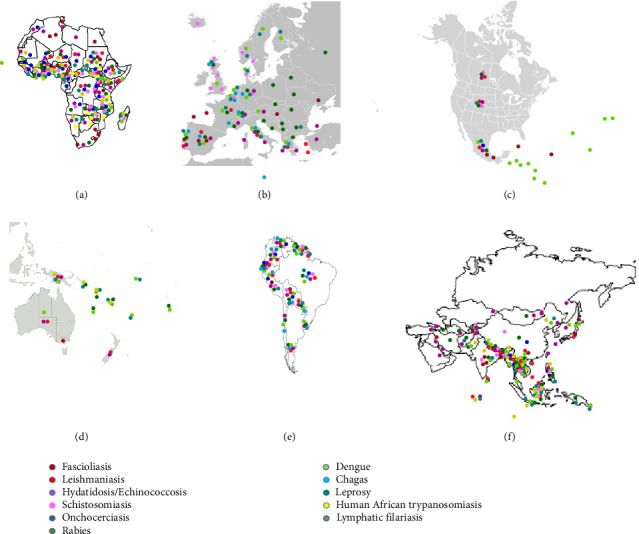
Geographical distribution of selected tropical diseases affecting female fertility.

However, it is worth highlighting that 13 primary NTDs have been explicitly prioritised based on their widespread occurrence (Figure 2; Table 2) and the significant burden they impose on world health (Table 1; Table 3). These NTDs include several pathogens, including helminths, protozoa, and tropical bacteria (Table 1) [6, 36]. Consequently, NTDs have the highest incidence rates among communities (Figure 2) that possess little capacity to access imperative services, including women, children, ethnic minorities, and displaced individuals [63].

**Figure 3:**
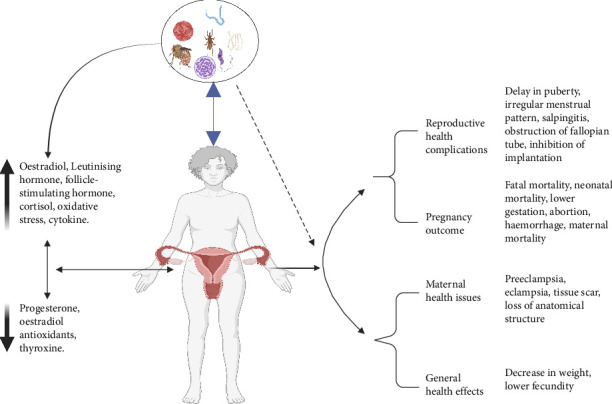
Effects of neglected tropical diseases (NTDs) on female fertility and the possible associated mechanisms. NTDs have been reported to suppress (black arrow) progesterone, estradiol (in some cases) antioxidants and thyroxine. On the other hand, although NTDs may elicit increased (brown arrow) oestradiol, luteinising hormone, follicle-stimulating hormone, cortisol, oxidative stress and cytokines, they also induce reproductive health complications, pregnancy outcome, maternal health issues and general health effects.

Frequently, women and girls bear a larger part of the burden associated with NTDs as a result of their disproportionate levels of poverty, illiteracy, poorer educational attainment, and social standing [64, 65]. A case-in-point that has been well documented is trachoma. It has been shown that female carriers tend to have more frequent contact with infected children compared to male carriers, resulting in a higher likelihood of contracting the infection themselves [66, 67]. Previous research has highlighted the tendency to underestimate and underreport NTDs in females, particularly schistosomiasis and lymphatic filariasis (LF). This is due to the diagnostic procedures for these diseases, which often necessitate women's compliance with activities that are culturally deemed inappropriate or taboo. These activities include the provision of urine or stool samples, as well as undergoing intimate physical examinations [6, 68]. The existing literature has extensively documented the disparities in the burden of NTDs across genders [69–71]. However, there is a dearth of studies investigating the impact of NTDs on female reproductive health. By using a gender lens to examine health seeking for certain NTDs, this review research sought to evaluate and annex the possible mechanisms (Figure 3; Table 3) and close this gap.

## 2. NTDs

**Figure 4:**
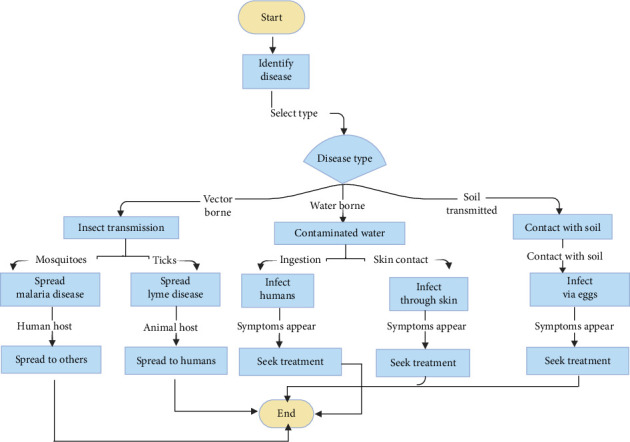
Schematic representation of selected causative organisms of neglected tropical diseases and their routes of transmission.

The NTDs include a category of infectious diseases. The causative agents encompass a physiologically diverse assortment, including protozoa that are transmitted by vectors, such as *Trypanosoma cruzi* [72], bacteria, specifically ocular serovars of *Chlamydia trachomatis* [73], filarial worms like *Onchocerca volvulus* [74], soil-transmitted helminths (STHs) [75] and two species of non-TB mycobacteria that are responsible for causing Buruli ulcer and leprosy, for which the mechanisms by which these infections are acquired are not yet fully understood [76]. NTDs may arise due to several causative agents, including parasitic organisms such as helminths and protozoa, as well as bacteria, fungi, ectoparasites and viruses (Figure 4). A collection of 13 illnesses accounts for about 57 million disability-adjusted life years lost annually [36, 77].

There is a gap in the knowledge and awareness of certain NTDs in some nations, and this expertise is progressively diminishing in other regions. There is also a substantial decline in competence seen in the domains of vector control, case management, pesticide management and veterinary aspects of public health [78]. Transmission occurs through various means, including flies [76, 79], fomites (e.g., skin cells, hair, clothing or bedding) [80], fingers (trachoma) [81], mosquitoes (dengue fever and filariasis) (Figure 4) [79], tsetse flies (sleeping sickness) [82], sandflies (leishmaniasis) [83], blackflies (onchocerciasis) [84] and snails [85]. Additionally, transmission can also occur through the faecal–oral route or via contaminated food products [86]. Additional control mechanisms and tools are required for nearly all NTDs, including those designated for elimination according to the 2012 London Declaration for NTDs and the 2013 World Health Assembly resolution (WHA 66.12). These mechanisms and tools encompass novel NTD drugs, vaccines and diagnostics, as well as vector control agents and strategies. The absence of these new instruments will render elimination unattainable [87].

### 2.1. Infertility and Female Reproduction

Female infertility is a prevalent factor that contributes to challenges in achieving successful reproduction. A significant proportion, around 50%, of couples seeking medical advice for infertility will present with a female spouse experiencing a reproductive health issue [88]. The prevalence of female infertility is around 37% among couples experiencing infertility [89]. Nevertheless, it should be noted that infertility is not exclusively attributed to women [90]. While there is a lack of accurate data on the worldwide prevalence of infertility [91], the occurrence of female infertility is increasing and ranges from 10% to 20% [92]. Approximately 15% of women globally may have either primary or secondary infertility [93]. According to Mascarenhas et al. [91], the reported incidence of primary infertility ranges from 0.6% to 3.4%, whereas the rate of secondary infertility ranges from 8.7% to 32.6%.

Understanding the aetiology of infertility is a crucial factor in identifying and describing women who are experiencing infertility [94, 95]. According to Malekshah et al. [96], variations in the causes of infertility are mostly attributed to disparities in cultural factors, social status, healthcare approaches, policies and environmental circumstances. Ovulation, pelvic inflammatory disease, tubal factor, intrauterine adhesions [97], advanced maternal age, high body mass index, age at first sexual intercourse, previous pelvic operations and psychological stress were identified as the primary risk factors contributing to female infertility [92].

The financial consequences of female infertility treatments are significant, resulting in a huge economic burden [98]. Nevertheless, it is crucial to remember that Aflatoonian et al. [99] recommended viewing infertility as a clinical manifestation rather than a disease. Couples who are undergoing in vitro fertility (IVF) treatment experience a significant amount of stress [100]. The implications of infertility include social effects, personal distress, mental health issues [3, 4] and sexual dysfunctions [5]. Marital disagreement often occurs among couples experiencing infertility, especially when they face the added burden of making medical choices [4]. The prevalence of clinical depression is noteworthy among infertile women, comparable to that observed in women diagnosed with heart disease or cancer [90]. Current research and clinical observations consistently suggest that the majority of individuals experiencing infertility do not encounter substantial psychological trauma. However, the utilisation of advanced medical techniques and the involvement of third parties in the reproductive process may heighten psychological distress during certain stages of treatment [101].

#### 2.1.1. Hydatidosis/Echinococcosis and Female Infertility

The zoonotic disease hydatidosis, sometimes referred to as *echinococcosis* (Table 1), is brought on by tapeworms belonging to the *Echinococcus* genus [102]. Although it mostly affects animals, this parasite illness may spread to people and cause serious health issues [103]. According to Beigh et al. [23], the definitive host harbours the adult tapeworm in their intestines, which excretes its eggs into the surrounding environment. The eggs hatch and the larvae travel to other organs, especially the liver and lungs, and feed on contaminated food or water the intermediate host consumes.

Jhim et al. [104] reported that hydatidosis may be difficult to diagnose since it often exhibits nonspecific symptoms. Nonetheless, hydatid illness is sporadic in the vaginal organs, with an incidence of about 0.5% [105]. The organs affected (the cyst's location) and the degree of infection determine the clinical signs in intermediate hosts, but often none are noticeable [102]. One symptom that may arise from an infection affecting the reproductive organs is stomach discomfort [106]. Hydatidosis may have an effect on female infertility (Table 1), which is one of the disease's lesser-known side effects [107]. Pelvic hydatidosis with bilateral fallopian tube involvement is a dreadful condition that adversely affects the fertility of young patients [108]. A pelvic hydatid cyst has been reported in an infertile young female who married at 14 and had not been pregnant ever [109].

Large Morgagni hydatid, an extremely rare cause of tubal torsion, has been reported to be associated with oxidative stress [40] which can affect the integrity of the ovaries and fertility. The causal relationship between oxidative stress and Morgagni hydatid is uncertain. Hydatid cysts may disrupt the endocrine system through the secretion of hormones that might potentially interfere with the regular functioning of the reproductive organs (Figure 3). The presence of this hormonal imbalance may lead to anomalies in the menstrual cycle and disruptions in ovulation, ultimately resulting in infertility (Figure 1) [107]. Hydatid cysts may also develop near the fallopian tubes or uterus, leading to mechanical blockage [106]. This may obstruct the transit of eggs and sperm, hence impeding fertilisation.

Although there have been several publications on echinococcosis, our understanding of the condition in pregnant women is limited due to its low occurrence during pregnancy, estimated to be about 1 in 20,000 to 30,000 cases [110]. Hydatid illness during pregnancy, although mostly lacking noticeable symptoms, might manifest as stomach discomfort, a tumour, or nonspecific abdominal symptoms [22]. The situation is very urgent and poses a significant risk to life since these patients are experiencing anaphylactic shock or abrupt death [111]. Pelvic hydatid disease may manifest as lower abdomen discomfort, disruptions in the menstrual cycle, an inability to conceive and symptoms arising from the compression of adjacent organs [112].

Pregnancy symptoms, such as moderate discomfort in the upper abdomen caused by reflux oesophagitis and feelings of nausea or vomiting, might make it difficult to identify comparable symptoms of abdominal/hepatic hydatid illness [113, 114]. Pregnancy often leads to back discomfort, which may also be a symptom of pelvic hydatid disease [115]. Pregnancy-induced reduction in cell-mediated immunity may promote the fast proliferation of parasites, causing hydatid cysts to significantly increase in size, resulting in symptoms or difficulties [116, 117]. The expanding uterus may exert pressure on hydatid cysts, leading to their rupture and causing severe anaphylactic shock, as well as the spread of daughter cysts. In addition, the increased pressure inside the abdomen that occurs during labour may be worsened by hydatid cysts and lead to the rupture of the uterus [118, 119]. Pelvic hydatid cysts may disrupt the typical progression of pregnancy by impeding the engagement of the presenting portion, blocking labour or triggering preterm labour [120]. Performing surgery to treat hydatid disease during the latter stages of pregnancy carries a higher likelihood of cyst rupture and may also trigger the onset of labour [121].

#### 2.1.2. Fasciola and Female Infertility

*Fasciola gigantica* and *Fasciola hepatica* commonly known as the liver fluke are the sources of fasciolosis, a parasitic worm illness also known as a plant-borne trematode zoonosis illness [122]. It is categorised as a NTD [17]. Fascioliasis manifests itself initially with little or no symptoms, including fever, malaise, stomach discomfort, gastrointestinal issues, urticaria, anaemia, jaundice and respiratory symptoms (Table 1) [17, 18]. However, mounting evidence has linked fascioliasis and infertility [39].

El-Khadrawy et al. [39] discovered a correlation between fascioliasis and infertility. The study observed that individuals with fascioliasis had bilateral smooth, inactive ovaries. Additionally, 28.9% and 38.9% of the subjects tested positive for fasciola using parasitological examination and enzyme-linked immunosorbent assay (ELISA), respectively. Research conducted by Biro-Sauveur et al. [123] found that fluke-infected heifers had a substantial delay of 39 days in the commencement of their first oestrus. Furthermore, López-Díaz et al. [38] found that heifers infected with flukes had significantly reduced levels of progesterone (P4) and oxidative stress. Compared to the control group, infected animals experienced a 39-day delay in the onset of the first oestrus. The infected group had substantially elevated serum concentrations of oestradiol (E2) compared to the control group, although P4 values were lower. The underdeveloped or absent corpora lutea are most likely to blame for fascioliasis' disruption of the blood's E2 level, which results in a lower-than-normal level of P4.

#### 2.1.3. Schistosomiasis and Female Infertility

A parasitic worm from the genus Schistosoma causes the tropical illness schistosomiasis, also known as bilharzia. The transmission cycle requires the introduction of excrement into surface water, certain freshwater snails as intermediary hosts and direct contact between humans and water [25]. Three primary species of schistosomes parasitise humans: *Schistosoma haematobium, Schistosoma mansoni* and *Schistosoma japonicum* [124]. Blood flukes, which are trematode worms from the genus *Schistosoma*, are the disease that causes schistosomiasis. It may manifest as either an acute or chronic condition [125]. There are 2 major forms of schistosomiasis—intestinal (*S. mansoni, S. japonicum, Schistosoma mekongi, S. guineensis,* and related *Schistosoma intercalatum)* and urogenital (*Schistosoma haematobium)* [126, 127]. Kokaliaris et al. [128] stated that the primary indicator of urogenital schistosomiasis is the presence of haematuria, which refers to blood in the urine. Urinary schistosomiasis emerges as a consequence of lesions present on the bladder wall, resulting in bloody urine (haematuria), chronic pain, anaemia, pollakisuria, proteinuria and dysuria [25]. This condition may also result in enduring and permanent effects, such as the inability to conceive.

Female genital schistosomiasis (FGS) induces discomfort and has been linked to stress, urinary incontinence, infertility and a heightened likelihood of abortion. Regrettably, it is possible that therapy may not effectively address these severe types of damage to the vaginal tract [129]. *S. haematobium,* which causes FGS, has a significant impact on women's reproductive health [41]. Ova inside the vesical plexus undergoes migration to the genital tract, resulting in the development of inflammatory lesions in the ovaries, fallopian tubes, cervix, vagina and vulva. Sandy patches in the lower vaginal tract are a clear indication of FGS. These patches are linked to developing new blood vessels and easily damaged mucous membranes, which may cause bleeding upon touch [41, 42].

Researchers have linked *Schistosoma haematobium* and *Schistosoma mansoni* to hypogonadism, delayed puberty (Figure 3), and primary and secondary infertility in both human and animal research [43, 130–134]. One study found a correlation between self-reported infertility and the presence of oestrogen-like metabolites during FGS [43]. Ribeiro et al. [44] discovered a drop in the serum levels of E2 in infected females but not in infected males (as anticipated given prior findings in men). Increased E2 explains male hypogonadism and the ensuing infertility, whereas lower-than-normal E2 levels in infected females amplify female infertility.

Both the human host and the schistosome parasite undergo significant oxidative stress due to the release of free radicals. Either the parasite itself during respiration or the immune response of the human host produces these radicals. This process leads to the production of ferrous iron (Fe2+) and toxic heme through the breakdown of haemoglobin (Hb) [135]. Remarkably, the schistosome parasite lacks effective mechanisms to cope with oxidative stress. However, despite this disadvantage, the worm has developed enduring strategies to thrive and inhabit the human host for as long as 3 decades without encountering significant consequences [136].

Infertility is thought to be associated with tissue scarring, inflammation and granulomatous responses caused by the release of proteolytic enzymes by the ova. This leads to mechanical obstruction, scar tissue formation and the loss of anatomical structures [59, 137–139]. It is thought that the development of this illness in the genital tract is caused by the buildup of ova and the inflammation that follows. This causes scar tissue, mechanical obstruction and damage to the body's structures [140].

FGS may also lead to endometritis, which is a condition that can result in female infertility. Urogenital schistosomiasis is a common and often undetected condition in those who are of reproductive age [44]. Lesions in the upper genital tract, which includes the uterus, fallopian tubes and ovaries, are less discernible by routine clinical examination [60]. Systematic histopathologic investigations conducted in regions affected by schistosomiasis have definitively shown the existence of schistosoma eggs and adult worms in the upper and lower female genital organs [141, 142]. In their study, Helling-Giese et al. [45] discovered a wide range of genital abnormalities in a group of Malawian women who were infected with *S. haematobium*. However, they observed that only sandy patches on the cervix and vaginal tumours seemed to be unique to FGS. Macroscopic lesions are characterised by certain histopathological patterns, and some kinds of lesions are limited to specific anatomical locations. The results have significant consequences for the reproductive and overall health of women. Further examination is required to explore the potential correlation between FGS and cervical cancer, as well as the transmission of HIV and FGS.

#### 2.1.4. LF and Female Infertility

Protozoan infections may lead to female infertility via local inflammatory processes or hormonal imbalances resulting from the infection (Figure 3) [61]. Evidence suggests that helminthic parasites, such as *Schistosoma haematobium*, can impact fertility by causing tubal occlusion, leading to infertility and ectopic pregnancy. Infection with *Schistosoma* sp. can also result in hormonal imbalances and dysregulation linked to infertility.

Residing in regions with high S. haematobium incidence in East Africa (Figure 2) was substantially linked to infertility [143]. Adult stages of the filarial worm *Wuchereria bancrofti*, the primary agent of LF, have been discovered in nodules or lymphatic vessels of the genital tract. This presence has been linked to the development of salpingitis, obstruction of the fallopian tubes and ectopic pregnancy [144–146]. Microfilariae of W. bancrofti and other filarial species like *Loa loa* and *Mansonella perstans* have been detected in the follicular fluid and cervicovaginal smears. However, the effect of their presence on fertility remains unclear [147–150]. A community-wide investigation demonstrated no impact of LF infection on fertility. However, a significant correlation was seen between having microfilaraemic and irregular menstrual patterns in women aged 30 years and older [151]. *L. loa* and *M. perstans* microfilaraemia may impact the hormonal system, causing delays in puberty, disruptions to the menstrual cycle and infertility in elderly individuals [152]. STH might affect fertility. A longitudinal study in Bolivia revealed that being infected with *Ascaris lumbricoides* was linked to earlier first births and shorter interbirth intervals, while hookworm infection was associated with delayed first pregnancy and longer interbirth intervals [153].

#### 2.1.5. Human African Trypanosomiasis and Female Infertility

Human African trypanosomiasis is a NTD found in sub-Saharan Africa, where the tsetse fly acts as the vector [154]. There are two types of the disease: one that progresses slowly in western and central Africa caused by *Trypanosoma brucei gambiense*, and one that advances more quickly in eastern and southern Africa (Figure 2) [155]. According to the literature, some protozoan parasites like *Trichomonas vaginalis* may lead to genital tract abnormalities, cervical neoplasia and unusual pelvic inflammations in women [58].

Pathogenic animal trypanosomes are responsible for prevalent livestock diseases that significantly affect the economies of several African nations [156]. Recent studies indicate that they induce various reproductive disorders in animals, such as degeneration of the hypothalamus, pituitary glands and gonads, leading to disruptions in hormone secretions and plasma concentrations essential for normal reproductive functions in both genders. Trypanosomiases in female animals result in severe vaginal lesions, temporary or chronic anoestrus, and irregular oestrous cycles. Trypanosomal-induced mortality during pregnancy, abnormal pregnancy, dystocia, abortion, early delivery, low birthweight, stillbirth, transplacental foetal infection, neonatal death and other harmful consequences on foetuses and progeny have been documented (Figures 1 and 3) [57].

Sekoni [57] stated that anterior pituitary (adenohypophysis) lesions have been seen in horses, lambs and dogs infected with *T. brucei*. Lesions and dysfunctions in the anterior pituitary, adrenal and thyroid glands were seen in male cattle infected with *T. vivax*. Female goats and Boran cows infected with *T. congolense* have shown elevated blood progesterone levels. Female goats with chronic *T. congolense* infection showed reduced plasma progesterone levels, whereas *T. vivax*-infected ewes had sustained a reduction of progesterone. Heifers and female goats infected with a certain condition showed increased levels of cortisol in their blood. Additionally, the infected heifers had a gradual reduction in thyroxine levels in their serum. Research on female goats showed a decrease in plasma progesterone, peak luteal progesterone and preovulatory oestradiol levels from the second to the fourth cycle after infection. The most continuous abnormal effect of trypanosomiasis on blood progesterone levels is a decrease or cessation of production, leading to the interruption of cyclicity in nonpregnant animals or abortion in pregnant animals.

#### 2.1.6. Onchocerciasis and Female Infertility

Blackflies, insects belonging to the genus *Simulium*, carry the disease onchocerciasis. The causative agent of this illness is the worm *Onchocerca volvulus* [157]. This disease was first identified during the arrival of explorers in Africa and the Arabian Peninsula. Subsequently, those afflicted with the illness were noted to undergo unexplained blindness, as well as the presence of scabies itch and the formation of nodular skin, often referred to as “kru kru” or “craw craw” in West Africa [158]. The disease is prevalent throughout a significant portion of tropical Africa and some regions of Central and South America, as well as Yemen (Figure 2). The vast majority (96%) of the estimated 122.9 million (Table 1) individuals at risk of the illness worldwide reside in sub-Saharan Africa (Figure 2), while Africa is home to 17.5 million of the estimated 17.7 million infected individuals [27].

Although there is a dearth of information on onchocerciasis and female infertility, some authors have cited both clinical and cultural evidence, suggesting that onchocerciasis may affect the female reproductive system. Microfilariae have been detected in gynaecologic smears [159] and vaginal irrigation specimens [160], providing clinical evidence. The possibility of onchocerciasis being transmitted inside the uterus has been suggested based on the detection of positive skin snips in newborns and infants [161]. In a retrospective study, Guderian et al. [28] examined the frequency of spontaneous abortions in an area of Ecuador where onchocerciasis is a serious problem. The study compared the number of abortions before and after treatment with ivermectin and also compared it to a similar region unaffected by the disease. The results indicated that the frequency of spontaneous abortions was linked to the change in the community's microfilariae load, suggesting a potential association between spontaneous abortions and *O. volvulus*. In the region where the disease is often seen, there was a noticeably higher occurrence of spontaneous abortions before the distribution of ivermectin compared to after the initiation of ivermectin therapy every 6 months. No significant change in the frequency of spontaneous abortions was observed in the nonendemic region over the same time frame.

#### 2.1.7. Chagas Disease and Female Infertility

*Trypanosoma cruzi* (*T. cruzi*) is the pathogen responsible for human Chagas disease, a prevalent disorder that affects around 16–18 million individuals in Latin American nations (Table 1). T. cruzi affects at least 2 million women in Latin America who are in the prime of their reproductive years [53]. *T. cruzi* is a type of single-celled organism that causes Chagas disease [162]. Chagas disease is a tropical illness that may be transmitted between animals and humans.

Multiple investigations have shown a correlation between Chagas illness and female infertility. Around 2 million women in Latin America who are of reproductive age have Chagas disease [53]. Chagas disease significantly reduces female fertility by impairing their reproductive capacity, maybe due to infection of the hormone-producing gland [163]. In mice or rats inoculated with different strains of *T. cruzi* (belonging to TcI, TcII, TcVI or undefined genotypes), maternal–foetal transmission of parasites was either not observed or only infrequently observed (by testing blood parasites, haemoculture or histological studies in offspring), either for a long time [164–167]) or just before or during gestation [49–51, 163, 168]. Congenital transmission of this kind seems to occur independently of placental parasite invasion. In contrast, TcI, TcIV or TcV chronically infected mice's progeny showed 33%–66% positive *polymerase chain reaction* (PCR) results in two previous investigations [52, 53]; however, congenital infection was not shown in these pups.

The studies on humans are rare, but several studies showed the potent role of *T. cruzi* in mice infertility. Acute infection with the protozoa *T. cruzi* totally impaired the reproduction of female mice by drastically reducing their fertility and inducing tremendous foetal death [163]. They described the following changes in infected mice compared with controls: lower gestation, reduce in fecundity and high utero and neonatal mortality. Their results showed that about 80% of infected mice were infertile, and in those that developed gestation, all embryos died. Mjihdi et al. [163] found that acute infection with the protozoa *Trypanosoma cruzi*, the agent responsible for Chagas disease in Latin America, completely hindered mice's ability to reproduce by significantly lowering their fertility and causing a significant number of foetal deaths. Almost 80% of the infected mice were rendered infertile. In contrast, in the mice that underwent gestation, the parasite invaded the placenta, resulting in ischaemia necrosis and elevated levels of tumour necrosis factor-α (TNF-α) production in the mother [169].

Infertility of mice acutely infected with *T. cruzi* arises from a defect occurring after mating and before implantation [170]. In addition, *T. cruzi* infection does not affect the yield of primary oocytes, oocyte maturation, ovulation, fertilisation and first cleavage of the zygote [170, 171]. A flawed embryonic development occurring between fertilisation and implantation is the cause of infertility in mice with acute *T. cruzi* infection [170].

Acute *T. cruzi* infection is associated with anoestrus in experimental mice [172]. The mechanisms through which *T. cruzi* infection has adverse effects on fertility are currently not known, but some possible mechanisms are suggested, e.g., infection of hormone-producing glands, parasite invasion of the placenta, overproduction of inflammatory cytokines (TNF-α) in the oviducts and/or uterine horns and inhibition of implantation and cell division. Moreover, parasite burden and strains of *T. cruzi* might also influence the outcome of infection on fertility and reproduction disorders [170, 171]. Since most cases of this kind of infection occur in childhood or early adulthood, the majority of infected adult people are in the chronic phase of the illness [173]. Presently, there is a paucity of data to support the notion; it is less likely to provide proof of a relationship between *T. cruzi* acute infection and female infertility.

#### 2.1.8. Dengue and Female Infertility

Dengue is a significant public health issue in tropical and subtropical regions, resulting in around 100 million infections per year (Table 1) and 25,000 fatalities globally [31]. Four serotypes of the *Flavivirus* genus are responsible for dengue fever, and *Aedes aegypti* mosquitoes spread it. According to Belinato et al. [174], *Aedes aegypti* (L.) (Diptera: Culicidae) is a mosquito species that transmits the viral disease dengue. Although dengue is widespread in tropical regions (Figure 2) [175], there is a scarcity of research on its impact on female infertility.

Approximately 50% of the global population is susceptible (Table 1), as stated by the World Health Organisation (WHO). There was a higher incidence of dengue cases in females compared to men [176]. Studies have shown that contracting dengue during pregnancy may lead to the development of preeclampsia, eclampsia, haemorrhage and maternal mortality. However, there is no evidence to suggest that it is linked to the incidence of foetal deformities [46–48]. Research conducted in Brazil found that the prevalence of dengue among pregnant women has mirrored the pattern of the illness in the whole population, with a larger number of suspected cases during years with outbreaks. The prevalence of dengue in pregnant women surpasses the prevalence in the overall population across all geographical locations and periods. The rising occurrence of this phenomenon may be linked to the growing need for healthcare services among pregnant women who experience symptoms of the condition. Additionally, the improved provision of healthcare services specifically tailored for pregnant women, who are regarded as a distinct category in terms of medical treatment, may contribute to this trend [177, 178].

#### 2.1.9. Leishmaniasis and Female Infertility

Leishmaniasis is a global disease that affects both humans and animals. Protozoans, specifically those from the genus *Leishmania,* are a type of single-celled organism that causes it [179]. There are around 30 species involved, with 21 of them capable of causing diseases in humans [180] worldwide (Figure 2). The illness is presently recognised as a significant global public health issue [180] due to a recent rise in the number of cases. Third, in disease burden among WHO NTDs, leishmania's worldwide frequency has more than quadrupled in the last several decades alone [181]. Approximately 12–15 million individuals are affected in 98 countries (Table 2), with an estimated annual incidence of 0.7–1 million infections [21]. Leishmaniasis is prevalent in several regions around the globe, including the tropics and subtropics of Asia, the Middle East, Southern Europe, Mexico, Central America and South America (Figure 2, Table 2) [182].

*Leishmania* spp. are intracellular parasites that cause a spectrum of human diseases called leishmaniasis, including cutaneous (CL), mucocutaneous (ML) and visceral leishmaniasis (VL) as prominent forms [179, 183]. The predominant mode of transmission is the bite of infected sandflies, although new means of dissemination have been described recently: organ transplants, blood transfusions, contaminated cutting objects, and sexual and vertical forms [184, 185]. In a study conducted by Sánchez et al. [20], it was shown that leishmaniasis may adversely impact reproductive and foetal parameters in female mice.

Leishmaniasis is a parasitic infection that can cause oxidative stress in the host [186, 187]. The female reproductive organs are not immune to the effects of oxidative stress, and it can contribute to female infertility [188]. According to different research types, antioxidants like catalase and superoxide dismutase (SOD) shield oocytes from oxidative harm during folliculogenesis [188]. Limited information exists on the impact of this parasitic infection on reproductive parameters and pregnancy outcomes in both affected humans and animals.

Sánchez et al. [20] assess the impact of long-term CL leishmaniasis induced by *Leishmania amazonensis* on reproductive and foetal factors using a female mouse model. During the trial, clinical parameters were observed and recorded during the premating and gestational phases. Female mice were euthanised on the 19th day of gestation. At this time, the foetuses were weighed and their length was measured. Also, any instances of embryonic resorption and foetal mortality were documented. In the infected group, they recorded five instances of foetal fatalities and three instances of embryonic resorptions. Moreover, the infected group had a decline in fertility. The offspring of infected females exhibited decreased weight compared to the control group, while the infected group also showed a reduction in foetal length.

Women of all ages are susceptible to the illness, but leishmaniasis infection during pregnancy, especially the visceral form, is severe and linked to the transfer of the disease to the foetus and foetal mortality (Figure 3) [189, 190]. Pregnant women may have an increased vulnerability to infection because of the alterations in cellular immunity that take place during pregnancy [191].

#### 2.1.10. Leprosy and Female Infertility

*Mycobacterium leprae* or *Mycobacterium lepromatosis* are two long-lasting bacterial diseases that can cause leprosy, also known as Hansen's disease (HD) [192]. It has been shown that leprosy infections result in detrimental effects on the respiratory system, skin, eyes and nerves (Table 1). This may lead to complications such as the inability to perceive pain and the loss of limbs owing to repeated injuries or infections from unnoticed wounds [193]. Data from Neena et al. [55] and Smith et al. [194] suggest that *Mycobacterium leprae* infection with leprosy reduces fertility in some people who have the disease. Worldwide, about 35%–37% of newly reported leprosy cases are female (Table 1). However, several nations have demonstrated a low number of infections in women, perhaps due to underdiagnosis in females [34]. Furthermore, it was shown that around 54% of female patients diagnosed with leprosy had infertility, along with noticeable monthly irregularities [195, 196].

The correlation between leprosy and female infertility is not as well established as in men, where the disease's scarring of the testes is well-recognised as a common cause of infertility [197]. A study conducted in India has documented a decrease in the number of children born to mothers with leprosy [54]. However, the study's cohort consisted of individuals from Leprosarium, which makes it challenging to distinguish the impact of infertility from the consequences of being close to potentially infertile males and the higher utilisation of contraceptives in hospital environments. There is less information about women's awareness of the impact of leprosy on future offspring. Due to the persistent belief in some communities that leprosy is an inherited illness, some women may choose to refrain from becoming pregnant [198].

There is a dearth of studies that have examined ovarian function evaluation in female Lepromatous. In the few studies that did include histopathology, the ovaries were not shown to be affected [199–201]. According to Khanna et al. [202], they observed a correlation between multibacillary (MB) leprosy and menstruation abnormalities, as well as an increase in gonadotropin hormones, which suggests malfunction of the ovaries. When there are no other causes like anaemia and TB, MB leprosy may cause monthly abnormalities and an increase in gonadotropin hormones. Ovarian dysfunction may be a result of the increased autoimmunity in MB leprosy. In a prior investigation, Neena et al. [55] observed that 30% of women in their reproductive years around had menstrual abnormalities, which often occurred after the beginning of leprosy. Additionally, these women had considerably elevated levels of luteinising hormone (LH) and follicle-stimulating hormone (FSH).

Although lepra cells have been seen in the endometrium, fallopian tubes and vaginal mucosa of female patients with leprosy, it has been shown that leprosy does not lead to infertility [203, 204]. Hardas et al. [205] observed that, whereas pregnancy modifies the progression of leprosy, leprosy does not impact the menstrual cycle or fertility. Surprisingly, the endometrial biopsy conducted during the premenstrual period revealed a secretory phase in only 18% of participants in the research, suggesting the presence of a hormonal imbalance. Sharma et al. [206] found that leprosy does not have a direct impact on menarche, menstrual cycle, fertility or menopause. However, they observed that 10% of their patients experienced primary infertility, which is potentially higher than the reported infertility rate of 6% in the general population of India [207]. Conversely, Flegar et al. [208] discovered that 54% of female leprosy patients were unable to conceive, whereas King and Marks [195] documented significant monthly irregularities in leprosy patients. Pradhan et al. [56] documented menstrual irregularities in individuals with leprosy and found that initiating treatment early helped mitigate this issue. Nevertheless, he refrained from making any remarks about the reproductive status of his patients.

#### 2.1.11. Rabies and Female Infertility

Rabies is a fatal illness that may be transmitted between animals and humans. The most well-known *lyssavirus* in this group is the rabies virus (RABV) [209]. RABV is a persistent and lethal brain illness that affects both humans and other animals. According to Brunker and Mollentze [210], it spreads through bites or scratches from the saliva of rabid animals. Human rabies viral infections occur when animal bites or scratches expose muscle tissue to animal saliva harbouring the RABV [211]. Rabies poses a danger for maternal mortality and an unknown harm to the foetus, and it is linked to a 100% fatality rate among all infectious diseases [212, 213].

Based on previous research and the case study by Müller-Holve et al. [214], the likelihood of rabies being transmitted from mother to foetus via the placenta in humans is considered very unlikely. Diagnosing rabies during pregnancy is rather uncommon. As far as we know, the literature has recorded five occurrences in which newborn infants survived [214, 215]. The study conducted by Wu et al. [29] showed that the vaccination, administered in three doses without any adjuvant, resulted in infertility in more than 80% of the mice whether given in live form (8 out of 10 mice) or inactivated form (13 out of 14 mice). In contrast, the control group, which did not receive the vaccine, had a conception rate of 100% (10 out of 10 mice). Their investigation revealed that the endometrium in the uterus of treated female mice was in a condition of reproductive quiescence or inactivity.

## 3. Discussion

One prominent result of infertility is the rise in NTDs. Several causal agents, including parasitic organisms, helminths, and protozoa, as well as bacteria, fungus, ectoparasites, and viruses, can lead to NTDs. We investigated in this research the recorded effects of NTDs on female infertility (Figure 1). Usually known as echinococcosis, the zoonotic illness hydatidosis is caused by tapeworms of the Echinococcus genus [102]. Although echinococcosis has been the subject of multiple studies, its low frequency during pregnancy means that our knowledge of the illness in pregnant women is scant. Lower abdominal pain, changes in the menstrual cycle, infertility and symptoms resulting from the compression of surrounding organs can all show up as pelvic hydatid disease [112]. First presenting with few or no symptoms, fascioliasis causes fever, malaise, stomach pain, gastrointestinal problems, urticaria, anaemia, jaundice and respiratory problems. Still, growing data have connected fascioliasis and infertility to each other [39].

In both human and animal studies, researchers have also connected Schistosoma haematobium and Schistosoma mansoni to hypogonadism, delayed puberty, and primary and secondary infertility [132–134]. Tissue scarring, inflammation and granulomatous reactions brought on by the ova's proteolytic enzyme release are believed to be linked causes of infertility. Mechanical blockage, scar tissue development and anatomical structural loss follow from this [139]. Additionally causing endometritis, a disorder that could cause female infertility, is FGS. Frequent and usually unnoticed in persons of reproductive age, urogenital schistosomiasis is a frequent disease [44]. Through local inflammatory processes or hormonal abnormalities brought on by the infection, protozoan infections might cause female infertility [61]. Helminthic parasites, including Schistosoma haematobium, have been linked to tubal occlusion, which results in infertility and ectopic pregnancy and hence affects fertility. Certain protozoan parasites, including Trichomonas vaginalis, can cause atypical pelvic inflammations in women, cervical neoplasia and abnormalities of the genital system [58]. Documented have been trypanosomal-induced mortality during pregnancy, aberrant pregnancy, dystocia, abortion, early delivery, low birthweight, stillbirth, transplacental foetal infection, neonatal death and other detrimental effects on foetuses and offspring [57].

Though little is known about onchocerciasis and female infertility, some writers have highlighted clinical and cultural data, indicating that onchocerciasis could have an impact on the female reproductive system. Clinical evidence has come from microfilariae seen in vaginal irrigation specimens [160] and gynaecologic smears [159]. Based on the discovery of positive skin snips in neonates and infants, it is hypothesised that onchocerciasis might be passed inside the uterus [161]. Chagas disease and female infertility have been linked according to several studies. Although human studies are uncommon, multiple investigations showed the strong influence of T. cruzi in mouse infertility. By greatly lowering their fertility and causing significant foetal mortality, acute infection with the protozoa T. cruzi altogether prevented the reproduction of female mice [163]. While dengue is common in tropical areas [175], little is known about how it affects female infertility. Research has indicated that acquiring dengue while pregnant could cause preeclampsia, eclampsia, haemorrhage and mother death [48] do not find any data, though, linking it to the frequency of foetal anomalies. The effects of this parasite infection on reproductive parameters and pregnancy outcomes in both afflicted people and animals are not well known.

Although leishmaniasis infection during pregnancy, especially the visceral type, is severe and connected to the transmission of the disease to the foetus and foetal death, women of all ages are susceptible to the disease [189, 190]. The changes in cellular immunity seen during pregnancy may make pregnant women more vulnerable to infection [191]. Unlike in males, where the disease's scarring of the testes is well-known as a prevalent cause of infertility, the relationship between leprous and female infertility is not as clear-cut. The study's cohort included people from Leprosarium, and hence, it is difficult to separate the effects of infertility from the repercussions of being near possibly infertile men and the increased use of contraceptives in hospital settings. While lepra cells have been seen in the endometrium, fallopian tubes, and vaginal mucosa of female leprous patients, it has been demonstrated that lepra does not cause infertility [204]. Linked to a 100% fatality rate among all infectious illnesses, rabies poses a threat for mother death and an unknown damage to the unborn [213]. The case study [214] and other studies suggest that the placenta is exceedingly unlikely to transfer rabies from mother to foetus in humans. Pregnancy-related rabies diagnosis is quite rare.

## 4. Current Drug Therapies for the Treatment of NTDs

Historically, NTDs have been overlooked by the pharmaceutical sector and public health initiatives in general. Most people with NTDs deal with poor sanitation, limited nutrition and inadequate healthcare. Therapy is not affordable for them regardless of its availability [216]. Important weapons for the prevention and treatment of NTDs are pharmaceuticals, vaccinations, diagnostic technologies and vector control strategies. Unlike other illnesses, the limited progress of novel treatments for NTDs points to a lack of innovation. Though just 13 (0.93%) were dedicated for NTDs [217], 1493 creative drugs were licensed between 1975 and 1999. The conditions did not much improve in the next decade; of the 850 new therapeutic products registered between 2000 and 2011, just 5 (0.59%) were allocated for NTDs, all classified as new indications or formulations of existing medications; none were novel chemical entities [218]. Comprising 1.65% of the 4006 Phase I studies overall, 66 new items progressed to Phase I clinical trials for the prevention or treatment of NTDs between 2000 and 2014.

Mass drug administration (MDA) approaches in large patient populations allow NTDs—where judged safe—to be treated [219]. Standardised dosages are used; a dosage pole can be used to find a person's height so that the required dosage may be computed [220]. Furthermore, numerous more NTDs call for more sophisticated or costly diagnostic tests, and the impacted people usually need individualised therapy [221]. Fundamental to the preventive chemotherapy strategy advocated by the WHO for the eradication of five of the most widespread NTDs—trachoma, STHs, schistosomiasis, LF, and onchocerciasis—MDA, whereby at-risk populations receive treatment routinely without individual diagnosis.

Although there are exceptions, such the insufficiency of benzimidazoles alone for the treatment of whipworm [222], therapeutic interventions for trachoma, the three main STHs (roundworm, whipworm, and hookworm), and schistosomiasis are generally effective and often curative. Though the therapeutic options for these NTDs are limited and may be vulnerable to the development of drug resistance, there is measured optimism that should MDA be carried out with enough frequency, coverage and duration, and the WHO's 2030 elimination objectives can be reached [223–225]. Likewise, although therapies for LF are not usually judged curative, they are adept in greatly reducing the microfilarial offspring (the stages transmitted to mosquito vectors) [226, 227] of adult filariae (macrofilariae) and may exhibit partial anti-macrofilarial effects [228]. As such, these treatments are seen as consistent with elimination as a public health issue (EPHP) [229].

Though reports of suboptimal responses in Ghana [230], a standard dosage of ivermectin (150 μg/kg), necessary for onchocerciasis MDA, effectively eliminates the microfilarial skin-dwelling transmission stages of adult Onchocerca volvulus to blackfly vectors and induces a temporary sterilising (embryostatic) effect [231]. Ivermectin needs multiple rounds of mass medication delivery and has only little efficacy in eradicating adult worms [232]. Mathematical models of transmission dynamics including pharmacodynamics imply that annual ivermectin mass medication administration alone may be insufficient for attaining eradication in areas with strong transmission [229, 233]. Prior to ivermectin mass medication administration for onchocerciasis starting in West Africa in the late 1980s, transmission rates in 50%–75% of endemic communities were categorised as either extremely high (holoendemic) or high (hyperendemic) [234].

A big first step towards eradicating onchocerciasis [235] was the 2018 approval of moxidectin as a therapy [236]. Like ivermectin, moxidectin is a macrocyclic lactone marked by improved pharmacokinetics and pharmacodynamics that progressively lower microfilariae to low levels over a prolonged period [237, 238]. Moxidectin will just be one component of the more comprehensive plan required to reach general onchocerciasis eradication. Giving macrocyclic lactones to persons who are also highly infected with Loa loa [233], a filarial parasite widespread in central African woodlands raises safety questions. Considered a safer choice for patients infected with L. loa, which does not contain Wolbachia, tetracycline antibiotics show macrofilaricidal efficiency by eradicating Wolbachia endosymbionts from O. volvulus [239, 240]. If shorter treatment regimens are as effective as current options (e.g., doxycycline, requiring 4–6 weeks of daily administration), these indicate important adjuvant or alternative therapies to macrocyclic lactones and are probably going to see increased usage.

Furthermore, recent developments in the treatment repertory for other NTDs include effective, and for a long period, IDA therapy—which comprises ivermectin, diethylcarbamazine (DEC) and albendazole—lowers microfilaraemia. In the fight against LF [241, 242], this marks a significant progress. Concerns over the safety of DEC treatment in Africa, where many individuals have both DEC and onchocerciasis, are causing delayed development though [241]. Alone benzimidazoles are not as efficient against whipworm as combinations of well-known antiparasitic medications [243, 244]. Important components of a worldwide strategy to make preventive chemotherapy for neglected tropical illnesses better are shown by these examples: developing new drugs and employing old ones in innovative ways [233, 245, 246]. The continuous evolution of drugs provides protection against the possibility of growing drug resistance, a particularly alarming problem for diseases like schistosomiasis, which depend just on one therapy option [247].

## 5. Conclusion and Future Perspective

In essence, NTDs reduce female fertility via dysregulation of oxidative stress and cytokines and therefore upsetting the endocrine system. They can also affect the ovarian histomorphology, which causes female infertility. For the most often occurring NTDs, coadministering medications could have advantages and help prevent female infertility brought on by NTD infection. Effective utilisation of money and other resources to deliver development programmes is crucial as the development of medicines for NTDs is mostly driven by the unmet medical needs reported by the global health community and without the chance of major financial returns on investment. More clinical research is advised, nevertheless, to show the likely processes connected with NTDs and female infertility [152].

## Figures and Tables

**Table 1:** Primary neglected tropical diseases (NTDs) prioritised based on widespread occurrenc**e**.

Study	Disease condition	Alternative name	Prevalence (%)	Pathophysiology	Causative agent
[17, 18]	Fascioliasis	Liver fluke	4.5	Fever, malaise, stomach discomfort, gastrointestinal issues, urticaria, anaemia, jaundice, respiratory symptoms and weight loss in children	Helminth
[19–21]	Leishmaniasis	Aleppo boil, Aleppo button, Aleppo evil, Baghdad boil, Biskra button and Biskra nodule	0.15–0.18	Enlarged spleen and liver	Protozoa
[22–24]	Cystic echinococcosis	Hydatidosis	5–10	The size and location of the cyst determine the outcome. May result in lower abdomen discomfort, harm to the liver, lungs and brain	Helminth
[25, 26]	Schistosomiasis	Snail fever	3.3	Stress, urinary incontinence, infertility, inflammatory lesions in the ovaries, fallopian tubes, cervix, vagina and vulva. Enlargement of the liver and spleen	Helminth
[27, 28]	Onchocerciasis	River blindness	0.28	Intense itching, rashes and blindness	Helminth
[29, 30]	Rabies		0.0002	Severe inflammation of the brain and spinal cord, death	Virus
[31, 32]	Dengue		0.16	Platelet depletion and haemorrhage leading to death due to haemorrhagic dengue fever	Virus
Mondiale de la Santé and [33]	American trypanosomiasis	Chagas disease	0.24	Cardiomyopathy	Protozoa
[34]	Leprosy	Hansen disease	0.002	Nerve damage and limb disfigurement	Bacteria
[35]	Human African trypanosomiasis	Sleeping sickness	0.9	Neurologic problems and death	Protozoa
World Health Organisation, 2013	Lymphatic filariasis	Elephantiasis	1.0	Extreme swelling of the limbs	Helminth

**Table 2:** Continents with the incidence of neglected tropical disease**s**.

Neglected tropical diseases	Asia	Europe	Africa	South America	North America	Oceania
Fascioliasis	Iran	Spain	Egypt	Bolivia	Mexico	Australia
	China	France	Algeria	Peru	Cuba	
	Vietnam	Portugal	Angola	Ecuador	Puerto Rico	
	South Korea	Tajik Republic	Cape Verde			
	Japan	Turkey	Ethiopia			
	India		Ghana			
	Nepal		Morocco			
	Bangladesh		Nigeria			
	Myanmar		Senega			
	Thailand		South Africa			
	Philippines		Tanzania			
	North Korea		Tunisia			

Leishmaniasis	Afghanistan, Iran, Iraq, Syria, Saudi Arabia, Yemen, Pakistan, India, Nepal, Bangladesh, Sri Lanka, Uzbekistan, Tajikistan, Turkmenistan, Kazakhstan. Southeast Asia: Thailand and Myanmar	Spain, Portugal, Greece, Italy, France, Cyprus, Turkey	Sudan, Ethiopia, Kenya, Somalia, Uganda, South Sudan, Democratic Republic of the Congo, Chad, Mali, Nigeria, Cameroon, Central African Republic, Algeria, Libya, Morocco, Tunisia	Brazil, Colombia, Peru, Bolivia, Venezuela, Ecuador, Paraguay, Argentina, Guyana, Suriname	Mexico, United States, Canada	Papua New Guinea, Australia, New Zealand

Hydatidosis/Echinococcosis	China, Mongolia, Kazakhstan, Kyrgyzstan, Uzbekistan, Turkmenistan, Afghanistan, Iran, Iraq, Turkey, Syria, Lebanon, Jordan, Israel, Saudi Arabia, Yemen, Oman, UAE, Qatar, Bahrain, Kuwait	Greece, Italy, Spain, Portugal, Turkey, Albania, Bulgaria, Romania, Kosovo, Bosnia and Herzegovina, Montenegro, North Macedonia, Serbia, Croatia, Slovenia	Morocco, Algeria, Tunisia, Libya, Egypt, Mauritania, Mali, Niger, Chad, Sudan, Ethiopia, Somalia, Kenya, Tanzania, Uganda, Rwanda, Burundi, Democratic Republic of the Congo, Angola, Zambia, Zimbabwe, Namibia, Botswana, South Africa	1. Argentina, Chile, Peru, Bolivia, Brazil, Colombia, Ecuador, Venezuela, Paraguay, Uruguay	United States, Canada, Mexico	Australia, New Zealand, Papua New Guinea

Schistosomiasis	China, Philippines, Indonesia, Cambodia, Laos, Vietnam, Myanmar, India, Bhutan, Bangladesh	France, Italy, Spain, Portugal, Greece, Cyprus, Germany, United Kingdom, Netherlands, Belgium, Switzerland, Austria	Nigeria, Democratic Republic of the Congo, Tanzania, Uganda, Kenya, Ethiopia, Sudan, Mozambique, Ghana, Ivory Coast (Côte d'Ivoire), Madagascar, Malawi, Zambia, Zimbabwe, Angola, Cameroon, Burundi, Rwanda, Sierra Leone, Togo	Brazil, Suriname, Venezuela, French Guiana, Ecuador, Bolivia, Peru, Colombia	The United States and Canada (In rare cases)	NILL

Onchocerciasis	Yemen, Saudi Arabia, Sudan, South Sudan	NILL	Nigeria, Democratic Republic of the Congo, Ethiopia, Sudan, Uganda, Ghana, Cameroon, Central African Republic, South Sudan, Chad, Côte d'Ivoire (Ivory Coast), Liberia, Sierra Leone, Togo, Guinea, Mali, Niger, Burkina Faso, Benin, Equatorial Guinea, Gabon, Guinea-Bissau, Mauritania, Senegal, Angola, Burundi, Kenya, Tanzania, Rwanda, Zambia	Brazil, Venezuela, Colombia, Ecuador, Peru, Bolivia, Guyana	Mexico	NILL

Rabies	India, China, The Philippines, Indonesia, Thailand, Vietnam, Bangladesh, Myanmar (Burma), Cambodia, Nepal, Sri Lanka, Malaysia, Pakistan, Laos, Mongolia, Bhutan, Afghanistan, Iran, Iraq, Saudi Arabia, United Arab Emirates (UAE)	Russia, Ukraine, Belarus, Poland, Romania, Bulgaria, Greece, Serbia, Hungary, Croatia, Bosnia and Herzegovina, Slovenia, Italy, Spain, Portugal, France, Germany, United Kingdom, Switzerland, Austria	Nigeria, Democratic Republic of the Congo, Ethiopia, Tanzania, South Africa, Kenya, Uganda, Sudan, Mozambique, Angola, Ghana, Zambia, Madagascar, Cameroon, Ivory Coast (Côte d'Ivoire), Burkina Faso, Niger, Mali, Malawi, Zimbabwe	Brazil, Argentina, Peru, Colombia, Venezuela, Ecuador, Bolivia, Paraguay, Chile, Uruguay, Guyana, Suriname	United States, Canada, Mexico	Papua New Guinea, Fiji, Solomon Islands, Vanuatu, New Caledonia, French Polynesia

Dengue	India, Thailand, Indonesia, Philippines, Vietnam, Malaysia, Bangladesh, Sri Lanka, Pakistan, Cambodia, Myanmar (Burma), Laos, Nepal, Maldives, Bhutan, Timor-Leste, Singapore, Brunei, Taiwan, China, Japan, South Korea	France, Spain, Italy, Portugal, Greece, Germany, United Kingdom, Netherlands, Switzerland, Belgium, Austria, Sweden, Denmark, Norway, Finland	Cape Verde, Mauritius, Réunion, Seychelles, Senegal, Kenya, Tanzania, Mozambique, Nigeria, Ghana, Uganda, Sudan, Ethiopia, Somalia, Madagascar	Brazil, Colombia, Peru, Venezuela, Ecuador, Bolivia, Paraguay, Argentina, Chile, Uruguay, Guyana, Suriname, French Guiana	Mexico, Belize, Guatemala, Honduras, El Salvador, Nicaragua, Costa Rica, Panama, Cuba, Jamaica, Haiti, Dominican Republic	Australia (particularly in northern regions), Papua New Guinea, Fiji, Samoa, Tonga, Vanuatu, New Caledonia, French Polynesia

Chagas	NILL	Spain, Italy, France, Switzerland, Germany, United Kingdom, Netherlands, Portugal, Greece, Belgium	NILL	Brazil, Argentina, Bolivia, Paraguay, Colombia, Venezuela, Peru, Ecuador, Chile, Uruguay	NILL	NILL
	India, Indonesia, Bangladesh, Myanmar (Burma), Nepal, Philippines, Sri Lanka, Vietnam, China, Thailand, Cambodia, Pakistan, Malaysia, Timor-Leste, Papua New Guinea, Laos	United Kingdom, France, Germany, Netherlands, Belgium, Italy, Spain, Portugal, Greece, Norway, Sweden, Denmark, Finland, Switzerland	Nigeria, Democratic Republic of the Congo, Ethiopia, Madagascar, Mozambique, Tanzania, Angola, Kenya, South Sudan, Cameroon, Chad, Guinea, Central African Republic, Uganda, Malawi, Zambia, Ghana, Sudan, Rwanda, Burundi	Brazil, Colombia, Peru, Venezuela, Bolivia, Paraguay, Ecuador, Suriname, Guyana, French Guiana	United States, Mexico	Papua New Guinea, Solomon Islands, Fiji, Vanuatu, Samoa, Marshall Islands, Palau, Micronesia, Kiribati, Tonga, Tuvalu, Nauru

Human African Trypanosomiasis	NILL	NILL	Uganda, Tanzania, Malawi, Zambia, Zimbabwe, Mozambique, Angola, Central African Republic, South Sudan, Kenya	NILL	NILL	NILL

Lymphatic filariasis	India, Bangladesh, Indonesia, Philippines, Myanmar (Burma), Nepal, Timor-Leste, Cambodia, Sri Lanka, Thailand, Vietnam, Malaysia, Papua New Guinea, Laos, Maldives, Bhutan, Pakistan, China, Solomon Islands, Kiribati, Marshall Islands, Micronesia, Palau, Vanuatu	NILL	Nigeria, Democratic Republic of the Congo, Burkina Faso, Tanzania, Uganda, Ghana, Ivory Coast (Côte d'Ivoire), Mozambique, Kenya, Benin, Madagascar, Malawi, Cameroon, Guinea, Niger, Togo, Senegal, Sudan, Ethiopia, Angola	NILL	NILL	Papua New Guinea, Solomon Islands, Vanuatu, Fiji, Samoa, Kiribati, Marshall Islands, Micronesia, Palau, Tonga, Tuvalu

**Table 3:** Associated mechanisms of neglected tropical diseases (NTDs) on female fertility.

Study	Disease condition	Study design	Associated mechanism and impact on female reproduction
[38, 39]	Fascioliasis	Cross-sectional	Hormonal imbalance and oxidative stress dysregulation; bilateral smooth, inactive ovaries
[20]	Leishmaniasis	Experimental	Foetal fatalities and embryonic resorptions
[40]	Cystic echinococcosis	Cross-sectional	Hormonal imbalance and oxidative stress dysregulation
[41–45]	Schistosomiasis	Cross-sectional	Hormonal imbalance and oxidative stress dysregulation; inflammatory lesions in the ovaries, fallopian tubes, cervix, vagina and vulva
[28]	Onchocerciasis	Retrospective	Spontaneous abortions
[46–48]	Dengue		Preeclampsia, eclampsia, haemorrhage and maternal mortality
[49–53]	American trypanosomiasis	Experimental	Hormonal imbalance and inflammation; foetal death
[54–56]	Leprosy	Cohort	Hormonal imbalance; decrease in the number of children
[57, 58]	Human African trypanosomiasis	Cohort	Hormonal imbalance; deformities of the genital tract, cervical neoplasia, tubal and atypical pelvic inflammations, foetal death, neonatal death
[44, 59–62]	Lymphatic filariasis	Cohort	Hormonal imbalance, inflammation; tubal occlusion, ectopic pregnancy

## Data Availability

The data that support the findings of this study are available from the corresponding author upon reasonable request.
